# Supporting Occupational Health and Safety Risk Assessment Skills: A Case Study of Five Companies

**DOI:** 10.3390/ijerph19031720

**Published:** 2022-02-02

**Authors:** Minna Rantala, Maria Lindholm, Sari Tappura

**Affiliations:** Center for Safety Management and Engineering, CSME, Faculty of Management and Business, Tampere University, 33014 Tampere, Finland; maria.lindholm@tuni.fi (M.L.); sari.tappura@tuni.fi (S.T.)

**Keywords:** development, expertise, occupational health and safety, risk assessment, skill, support, training

## Abstract

Financial burden due to poor occupational safety practices remains high although occupational health and safety (OHS) have improved in recent years. Conducting risk assessment is one way to improve OHS. Workplaces may not have sufficient expertise in risk assessment. The aim of this study was to identify the needed OHS risk assessment skills, current support in the workplaces and the ways to improve risk assessment skills. This study was conducted with the Delphi survey for OHS experts (*n* = 13) and with interviews (*n* = 41) in the case companies. OHS experts agreed that the most significant skills were for employees to identify hazards and minimize risks in one’s work; for supervisors to influence others with a good example; and for OHS experts to understand and manage the wholeness of safety practices and understand and manage the meaning, concepts, and criteria of risk assessment. The current main support methods were learning at work, training and written instructions. However, many of the interviewees felt that they had not received risk assessment training and that the support depended on their activity. Finally, the OHS experts determined that the most feasible ways to improve risk assessment skills were training, coaching and giving clear instructions. Likewise, the interviewees suggested various training methods. Based on these results, concrete development plans to improve risk assessment skills can be made.

## 1. Introduction

Even though occupational health and safety (OHS) have been developed and improved in recent years, almost two million employees die each year because of exposure to work-related risks [1]. The economic burden of poor OHS practices is estimated to be 3.9% of the global gross domestic product (GDP) (i.e., EUR 2680 billion) each year [2]. According to previous studies, health promotion programs and various interventions related to occupational safety and health seem to reduce absenteeism and healthcare costs, and they have the potential to achieve business benefits [3,4]. At workplaces based on Finnish Occupational Health and Safety Act [5] and European OSH legislation [6], employers are responsible for ensuring the safety and health of their employees, for preventing occupational accidents and diseases and for eliminating hazards stemming from the work environment and the work itself. One key responsibility of employers is to carry out hazard identification and risk assessment [5,7].

The OHS risk management process involves different concepts (e.g., hazard, hazardous events, consequences and risk) which can be defined in different ways, thus affecting the results of the risk assessment [8,9]. How risk assessors understand these concepts, the differences between them, and the process of risk assessment has a remarkable effect on the results and the success of the risk assessment [10,11,12]. Overall, the specific knowledge, experience, abilities, skills, attributes, values, attitudes, understanding and behaviors of the risk assessor may affect the OHS risk assessment results [13,14,15]. In addition to the risk assessor, initial assumptions, oversimplification, inaccuracy of consequence models, ill-defined data, as well as poor hazard identification, teamwork, chosen method and documentation may all affect the quality of a risk analysis [16,17,18]. Previous studies have found that risks remain unidentified or uncontrolled due to limitations in OHS risk assessment skills, processes and tools [11,12,19,20]. Risk assessments may also be omitted altogether or delayed [21]. In addition, the research suggests that insufficient guidance on how to use risk matrices may lead to variation in the quality of risk assessment [22].

Even though previous studies have recognized the effect of the risk assessor’s and the risk assessment team’s skills to conduct a risk assessment, not many studies have primarily focused on these assessment skills or on developing them. However, for example, Hrica and Eiter [23] identified critical competencies (i.e., general hazard knowledge, site-specific hazard knowledge and situational awareness) regarding hazard recognition, which is a part of OHS risk assessment skills. Previous research has revealed that when workers are trained by effective training methods, they are able to recognize more hazards and experience higher risk levels in similar cases than workers who have not received training [24]. Hazard identification can, for example, be developed through visual hazard identification exercises [25]. Brandt et al. [26] found that, in a participatory workshop, participants can discuss problems with their colleagues and might learn from others. Namian et al. [27] argued that both the type of education (low engagement or high engagement) and the training transfer effect (e.g., upper management engagement and supervisor support) are important. Achieving a better training transfer does not necessarily mean creating new processes, but more benefits accrue by integrating learning into the company’s existing process and reward systems [28].

OHS training often uses lectures and courses as teaching and training methods. In addition, novel approaches are being used, such as e-learning, online and distance learning, as well as computer-assisted teaching and virtual reality simulation [29]. Approaches requiring participation and commitment are generally considered to be more effective [30,31], and using examples pictures, and other illustrative methods has often been found to be more effective than using traditional lectures or reading material [32,33]

The need to develop risk assessment skills, and to do it based on one’s own initiative, is a widespread phenomenon in Finnish workplaces [21,34,35,36]. Previous research states that companies typically do not evaluate the risk assessment skills of risk assessors or the success of risk assessment [37]. Using the Occupational Health and Safety Act as framework, the aim of this study was to recognize what are the risk assessment skills of persons who carry out risk assessment related to OHS; how the skills are currently supported in the workplaces; and how the risk assessment skills can be improved. The results of the study can be used to make the OHS risk assessment more resource-efficient and better meet the needs of the workplace. Thus, risk assessment can reap the necessary benefits and serve as a tool for safety management and safety improvement. The practical contribution of the study consists of suggestions about what the risk assessment skills of employees, supervisors and OHS experts should include, and how to support risk assessment skills. 

The subsequent Materials and Methods subsection contains background information on the companies that cooperated and the research methods we used. The results obtained are presented grouped by theme. First, the OHS risk assessment skills of employees are discussed, in which the research method was the Delphi survey. Then, the results of the interviews on the current support for risk assessment skills are provided. Finally, the development of risk assessment skills was examined through both the Delphi survey and interviews. The discussion section considers the results obtained by different methods and compares them with previous research. In addition, further research and research limitations are presented at the end of the section. Finally, the main results and the obtained contribution are highlighted in these conclusions.

## 2. Materials and Methods

The aim of this study was descriptive in nature, and the phenomena of interest are contextual so that the researchers have limited influence on them [38]. In this mixed methods study [39], there were two different phases. In phase 1 (December 2020–January 2021), a Delphi survey [40,41] was conducted to explore what kind of risk assessment skills people in different positions need to have and how their risk assessment skills can be supported. In order to gain a deeper understanding of the current OHS risk assessment skills, in phase 2 (January–March 2021), a qualitative interview study was conducted using a semi-structured interview form [42]. The interviews were conducted in five Finnish companies from different industries: manufacturing, transportation and storage, electrical power generation, and another technical testing and analysis field. This qualitative and descriptive study has followed research ethical principles in accordance with the Finnish National Board on Research Integrity [43] and the General Data Protection Regulation [44]. Companies involved in this study committed to participate in interviews and surveys. Participants agreed to participate in the study after being informed of voluntary participation, anonymity and confidentiality.

### 2.1. Delphi Survey

The iterative Delphi survey is a method in which respondents evaluate their anonymous responses until a consensus is reached on the responses [40,41]. The Delphi survey consisted of three rounds in which the aim was to gather the views of OHS specialists and managers on what should be included in the risk assessment skills of the employee, the supervisor and the expert (e.g., the OHS representative or OHS manager). The request to participate included information about the study; instructions on how to participate in the survey; and the link to the survey. It was emailed to 17 individuals; 13 agreed, yielding a response rate of 76.5%. Three rounds of the Delphi survey were conducted. The response percentages for each round were 47.1% (*n* = 8) in the first; 70.6% (*n* = 12) in the second; and 76.5% (*n* = 13) in the third round. It was possible for the respondents to take part in the survey later, even if they had not been involved in the first round. In addition, it was assumed that OHS specialists and managers are, by definition, aware of the risk assessment principles. Their experience in occupational safety was on average 13 years (SD = 16). The respondents worked for industrial companies, research institutions and authorities (see summary in Table 1). The survey was created through Lime Survey.

Questions asked in the first round of the Delphi survey were “What is included in the risk assessment skills of (a) the employee, (b) the supervisor, and (c) the OHS expert?” and “How can risk assessment skills be supported?” Respondents (*n* = 8) answered open-ended questions and the length of the answers was not limited. Background information collected from the respondents included their name, the name of the company, the position in the organization (supervisor, specialist), work experience in the current position and work experience in similar positions. The name of the respondent was asked so that reminder emails could be targeted to people who had not yet responded. 

Researchers performed a qualitative analysis and the participants received feedback. The thematic categorization of the qualitative material of the first round was conducted by applying the open coding analysis approach [45]; the process identified four factors in the OHS risk assessment skills of employee, six factors in the OHS risk assessment skills of the supervisor and six factors in the OHS risk assessment skills of experts. In addition, responses to the question about how to support OHS risk assessment skills received 12 suggestions. In the second round of the survey, respondents (*n* = 12) commented on the categorized lists and explained their answers to open-ended questions.

After the second round, a quantitative analysis was performed using Microsoft (MS) Excel, and the participants again received feedback. In the third round of the survey, the respondents (*n* = 13) chose the four most important factors for employee’s OHS risk assessment skills, the five most important factors for supervisor’s and expert’s OHS risk assessment skills, and the eight ways to support OHS risk assessment skills. In addition, respondents chose the eight most feasible ways to support OHS risk assessment skills. If desired, respondents were allowed to choose fewer options for their response. The third-round answers were again analyzed using MS Excel and IBM SPSS. Before each round ended, participants were sent reminders to answer.

### 2.2. Semi-Structured Interviews

The companies were chosen based on their needs and interests to develop their risk assessment processes. There are some similarities and differences between the companies. Four of the companies are large, while one is medium sized, employing approximately 200 people. In addition, almost all companies employ subcontractors. One company operates only in Finland, while others have international operations, or they are part of larger international corporations. The companies involved in this study are stable, traditional Finnish companies, where attention is paid to employee persistence. The duration of employment of the persons interviewed at the company was on average 14 years (SD = 10). This study focused on the Finnish sites of these companies.

The interviews were conducted with members of the risk assessment team or with managers. The position of the interviewees varied widely depending on the company. In one company, the interviewees were chosen as a cross-section of the process from design to implementation, while in another, mainly managers and immediate supervisors were interviewed. Altogether, the interviewees’ positions were manager (*n* = 10), immediate supervisor (*n* = 15), employee (*n* = 20), and OHS manager or representative (*n* = 8). When describing the status of the interviewees, it must be considered that, for example, the OHS manager can also be the supervisor and that the OHS representative of employees is usually an employee themselves or an expert on certain tasks. They are classified here as OHS managers and representatives. Table 2 summarizes the distribution of the companies, the interviewees and the interviews. All the interviews were held via MS Teams. Interview types were divided into individual interviews (*n* = 32) and group interviews (*n* = 9), and the average duration of interview was 59 min, lasting between 38 and 96 min. The transcriptions resulted in 324 pages.

The semi-structured interview form included questions such as the following: “How is the conducting of a risk assessment instructed?”; “Have you/others involved in the risk assessment received orientation about the risk assessment? What kind?”; “How are individual employees instructed to assess the risks of their own duties?”; “How are risk assessment skills currently supported?”; and “In what ways could the risk assessment skills be improved?” A few questions had to be slightly modified for companies to suit their operations. The question “How are individual employees instructed to assess the risks of their own duties?” received an answer in 25 interviews. It was not answered if the interviewee was an expert with no subordinates, if there was time pressure due to the group interview, or the discussion was about guidance in general. One interviewee did not answer the questions “How are risk assessment skills currently supported?” and “In what way the risk assessment skills could be improved?” All the interviews were recorded except for one, when the interviewee did not want to be recorded. However, the notes from that interview were added to transcriptions. A total of three companies had both individual and group interviews; one company had only group interviews, and one had individual interviews. The duration of the interviews varied depending on whether it was a group or an individual interview. 

Transcriptions were analyzed using Atlas.ti version 9 software by applying the open-coding analysis approach [46] (i.e., written data were conceptualized into separate categories and given a name that represents or stands for it) [47] in order to identify the current risk assessment support and how to develop it. During the analysis, the codes that needed further processing were discussed together. Researchers coded one interview at a time, resulting in 482 codes. The researchers then checked the codes for consistency, removed unnecessary codes, and compiled similar codes, resulting in 409 codes. During the third round, the researchers categorized the codes into 104 themes. A thematic categorization of the data was conducted by using Whimsical SaaS because of its visuality for categorizing the themes that emerged. Questions with clear answers did not require thematic processing. Hence, two groups of codes were obtained, some of which were processed numerically and some through thematic design. Because there is more than one code categorized within a single theme, participants might have mentioned a particular theme more than once per interviewee. In other words, the number of respondents or interviews does not necessarily correlate with the number of themes. In addition, MS Excel was used as a support tool in the analysis. The questions and their occurrences in the interviews are presented in Appendix A, Table A1.

## 3. Results

The used risk assessment methods in the companies were checklists, the potential problems analysis (PPA) method and the task risk assessment completed either in the OHS system or with MS Excel and MS Word forms. The composition of the risk assessment team varied considerably depending on the company but usually comprised of the immediate supervisor and the employee(s), the OHS representative, and in some cases, safety/quality manager as well as the work experts.

### 3.1. Required OHS Risk Assessment Skills of Employee, Supervisor, OHS Expert

In the Delphi survey, the most significant OHS risk assessment skills of employees and supervisors were unanimously found. Identifying hazards and minimizing personal risks were the most significant skills for employees. Influencing others by their own example and attitude was seen as the most considerable skill for supervisors. The skill requirements of the experts were not as unanimously chosen as for the employees and supervisors. The two things considered to be the most significant OHS risk assessment skills were both understanding and managing the wholeness of safety practices and understanding and managing the meaning, concepts and criteria of risk assessment as well as the related workplace safety legislation.

#### 3.1.1. Employees’ Risk Assessment Skills 

**Figure 1:**
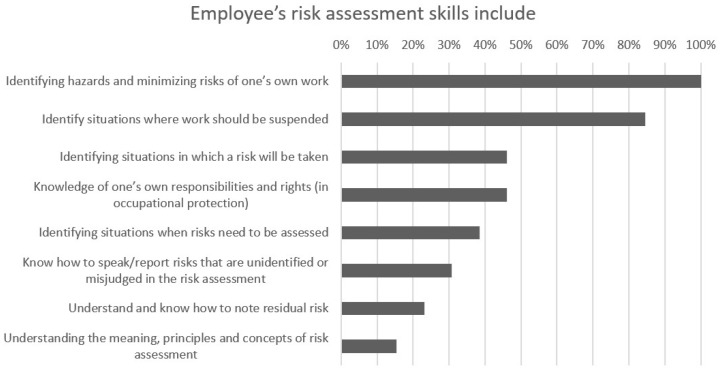
Opinions of occupational health specialists and managers on the most important risk assessment skills needed by the employee.

All respondents considered identifying risk factors in their own work and minimizing risks to be the most important factors in an employee’s OHS risk assessment skills. In addition, the theme “Identify situations where work should be suspended” was highlighted in the second round and was considered the second most important factor by over 80% of the respondents in the third round. “Identifying situations in which a risk will be taken” and “Knowledge of one’s own responsibilities and rights (in occupational protection)” were chosen as the third and fourth most important factors by 46% of the respondents, respectively. Employees are expected to have experience of the work and knowledge of the work-related hazards, which is why understanding the meaning, principles, and concepts of risk assessment was considered to be the least important factor in an employee’s OHS risk assessment skills. Two respondents chose only three options instead of four. Figure 1 shows the results given by occupational health specialists and managers on the requirements for employee risk assessment skills.

#### 3.1.2. Supervisors’ Risk Assessment Skills 

**Figure 2:**
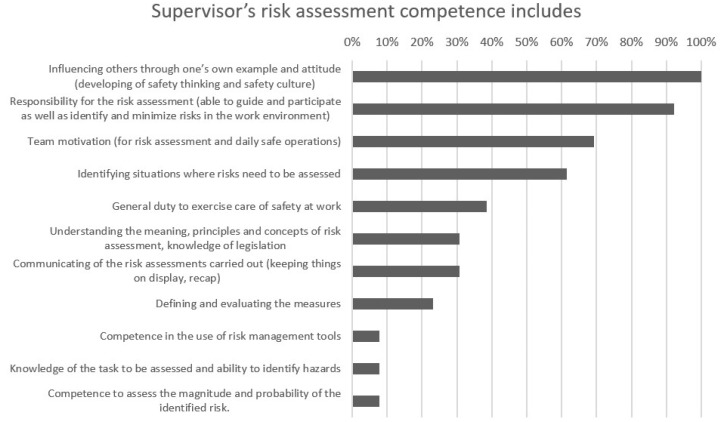
Opinions of occupational health specialists and managers on the most important risk assessment skills needed by the supervisor.

The most important factor in the supervisor’s OHS risk assessment skills was unanimously “influencing others through one’s own example and attitude (developing safety thinking and safety culture).” This option was added based on comments from the second round. The second most important factor, “Responsibility for risk assessment (able to guide and participate as well as identify and minimize risks in the work environment),” was chosen by 92% of the respondents. The third and fourth options also received more than 60% of choices. “Team motivation (for risk assessment and daily safe operations)” was added based on the second-round answers and was chosen by 69% of the respondents. “Identifying situations where risks need to be assessed” received the fourth highest score, as was chosen by 62% of the respondents. The fifth, “General duty to exercise care of safety at work,” received only 38% of the choices. The options which were only chosen once were “Competence in the use of risk management tools,” “Knowledge of the task to be assessed and ability to identify hazards,” and “Competence to assess the magnitude and probability of the identified risk.” Four respondents decided to choose only four factors instead of five. Figure 2 shows the results given by occupational health specialists and managers on the requirements for supervisor risk assessment skills.

#### 3.1.3. Experts’ Risk Assessment Skills

**Figure 3:**
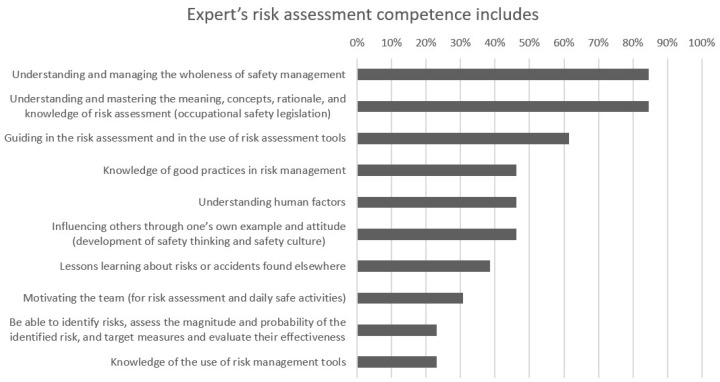
Opinions of the occupational health specialists and managers on the most important risk assessment skills needed by the expert.

The most important factors related to an expert’s risk assessment skills caused more variation than those of employees and supervisors. The two most important were “Understanding and managing the wholeness of safety practices” and “Understanding and mastering the meaning, concepts, rationale, and knowledge of risk assessment (occupational safety legislation),” which received 85% of the choices. “Guiding in the risk assessment and in the use of risk assessment tools” also received more than 60% of the choices. “Knowledge of good practices in risk management” and “Understanding human factors” were chosen as the fourth and fifth factors by 46% of the respondents. Indicating the variation of the answers, even the least voted factors received more than 20% of the points. For the experts, these factors were “Be able to identify risks, assess the magnitude and probability of the identified risk, and target measures and evaluate their effectiveness” and “Knowledge of the use of risk management tools.” Two respondents felt that four of the five factors were sufficient. Figure 3 shows the results given by occupational health specialists and managers on the requirements for expert risk assessment skills.

### 3.2. OHS Risk Assessment Skills Support at The Case Companies

Based on the interviews, learning at work and training were considered to be the most often used forms of orientation, mentioned in 56% and 59% of the interviews, respectively. Almost as many felt that they had not received risk assessment orientation. Written instructions were raised in all companies as a considerable way to guide the conduct of an OHS risk assessment. Perceiving the dangers in one’s own job was seen as a part of everyday activities. Some companies also use a so-called last-minute risk assessment, being mentioned in 29% of interviews. Lastly, in 63% of interviews, the interviewees felt that the support they received mainly depended on the employee’s own activity and that they needed to be able to either ask for it or apply it. The number of responses per interviews or mention per theme varied in each question. All results are presented in Appendix A, Table A1.

#### 3.2.1. Orientation Regarding Risk Assessment Process

There are different ways to support OHS risk assessment skills in the workplace. One way to support employees involved in OHS risk assessment is to have an orientation about how to carry it out. In OHS risk assessment orientation, the three most often mentioned themes in interview responses were almost equally divided between “Learning at work” in 56% of the interviews, “Training” in 59% of the interviews and “Employees feel that there was no orientation” in 56% of the interviews. It was felt that orientation can be obtained almost as much through on-the-job learning as through training:

*“Online training materials where all supervisors need to undergo these trainings. They are compulsory trainings.”*

*“I actually started using that program—and practiced myself.”*

It was generally pointed out in the responses that the skills were gained through work even prior to present-day orientations. Those who had worked less time felt that they had received more orientation. However, in an equal amount of the interviews, the respondents did not feel that they had received any orientation at all with regard to performing the risk assessment.:

*“I have not received any orientation with risk assessment in particular.”*

Responses to interviews in the “Other” category (20%) refer to a review of instructions prior to conducting a risk assessment, ongoing safety discussions and developing risk assessment models. Responses regarding orientation received similar answers in each company.

#### 3.2.2. Instructions to Perform a Risk Assessment and for Individual Employee to Assess the Risks of Their Own Duties

By instructing employees, support can be provided both to conducting the risk assessment and guiding the individual employee to identify the hazards associated with their own work. When asked how to conduct a risk assessment, in 56% of interviews, the risk assessment guidelines as well as the written instructions used in the company and their location were mentioned. Some companies also received instructions from their customers:

*“When you start doing a risk assessment, it goes how it is in this risk assessment form. It helps us, but it is not slavishly observed.*”

However, in 41% of the interviews, the respondents were uncertain regarding the instructions, the number of instructions was considered low, or the interviewee was unsure about the location of the guidelines or instructions. There was only one company where no one was unsure of guidance:

*“If I have to name a guide, I find it terribly difficult to say. But yes, I go to a safety management system where I don’t remember exactly what it says.“*

Training was mentioned in 34% of interviews. The interviewees felt that they had received guidance in various trainings, such as e-learning programs, educational days and video trainings. In addition, in 17% of interviews, guidance provided by a particular person, such as an OHS manager, or a leader of the risk assessment before each audit, was mentioned. In one interview, unwritten instructions were highlighted. At the corporate level, there were two themes—training and guidance provided by a particular person—that were not recognized in two companies. 

A total of 39 responses were received from the 25 interviews regarding the instructions of an individual employee concerning the assessment of the risks of their own work tasks. Nearly half of the respondents felt that assessing the risks of one’s own job was a daily activity:

*“A proactive approach to occupational safety [is needed], with the aim of making safety observations or reporting near misses, perhaps to achieve day-to-day foresight.*”

The second most common way to instruct assessing the risks of one’s own work is a last-minute risk or hazard assessment, which was mentioned in 29% of interviews. One of the companies used a paper memory card to perform a last-minute hazard assessment. In addition, one’s own activity was felt to be important in obtaining information in three responses. The oral work permit policy used in one company was also mentioned twice here as a guideline for assessing the risks of one’s own work. Two of the respondents felt that they were not introduced to how to assess the dangers of their own work. 

#### 3.2.3. Current Risk Assessment Skills Support

In 63% of interviews, the respondents thought that when they are active themselves, they also receive support. In other words, it was felt that support was available as long as it was requested. In addition, it was felt that individual initiative was needed to go through the guidelines:

*“But it [support] may not be actively offered directly. In that sense, … one has to be active if one wants to further develop it.”*

The next most usual way to support OHS risk assessment skills was various training sessions and guidelines, which were mentioned in 41% of interviews. In 29% of interviews, the respondents said that OHS risk assessment skills were not supported. In 22% of interviews, guided discussions, such as safety talks and meetings led by the supervisor, were seen as one way to support risk assessment skills. 

### 3.3. Development of OHS Risk Assessment Skills 

Based on the Delphi survey, no overwhelming feasible proposal was found for the development of OHS risk assessment skills. The two most frequently chosen development methods were the organization of training and coaching related to OHS risk assessment methods and clear instructions for the implementation of OHS risk assessments. Various training methods emerged in the interviews as the most significant way to develop OHS risk assessment skills.

#### 3.3.1. Feasible Ways to Support Risk Assessment Skills 

**Figure 4:**
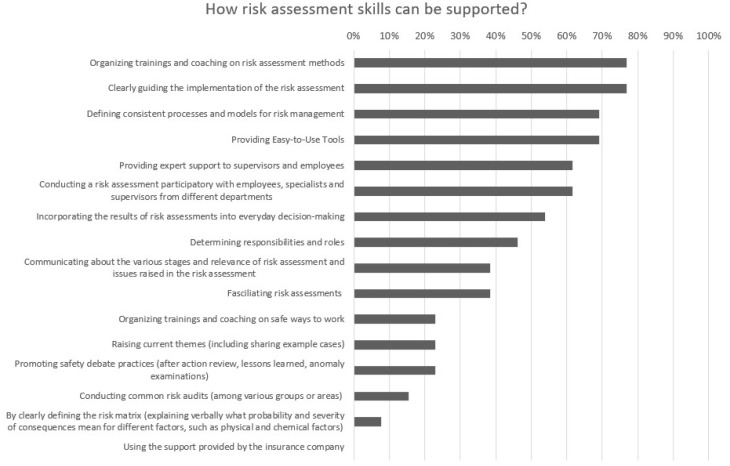
Opinions of occupational health specialists and managers for the most feasible ways to support risk assessment skills.

In the Delphi survey, the experts were asked to choose the eight most feasible ways to support OHS risk assessment skills. The question divided opinions and only seven of the respondents chose all eight options. The other six respondents either did not feel that they found any other feasible options on the list or were satisfied with fewer choices. The number of selected choices ranged from four to eight. The options receiving the most choices (77%) were “Organizing trainings and coaching on risk assessment methods” and “Clearly guiding the implementation of the risk assessment.” The other most viable forms of support that received more than 50% of the votes were “Defining consistent processes and models for risk management” (69%), “Providing easy-to-use tools” (69%), “Providing expert support to supervisors and employees” (62%), “Conducting a risk assessment participatory with employees, specialists, and supervisors from different departments” (62%) and “Incorporating the results of risk assessments into everyday decision-making” (54%). The choice for the most feasible way to support OHS risk assessment skills was not as unanimous as in the previous questions, which was partly due to the different risk assessment practices of companies. None of the respondents saw “Using support provided by an insurance company” as a the most feasible way to support OHS risk assessment skills. The form of support “By clearly defining the risk matrix (explaining verbally what probability and severity of consequences mean for different factors, such as physical and chemical factors)” was only chosen once. Figure 4 shows the results from the occupational health experts and supervisors on feasible ways to support risk assessment skills.

#### 3.3.2. Ideas for Developing OHS Risk Assessment Skills

Ideas for developing risk assessment skills varied more than the themes in the previous subsections. In 63% of interviews, participants mentioned training activities as the largest factor to improve risk assessment skills. The various training methods related to OHS risk assessment skills included, for example, training given by an outsider, video training, or training provided by a specific person working in the organization. In addition, the regular reviewing of the risk assessments, orientation of new employees and mentoring were mentioned:

*“It could be, for example, an online course that allows you to concretely identify certain dangers from pictures and in some way figure out the risks associated with them.”*

The next most common point was to increase the understanding of risk assessment (mentioned in 32% of the interview responses) and obtain different perspectives (mentioned in 24% of the interview responses):

*“It would be good to hear how to carry out a risk assessment elsewhere—whether it’s similar, whether similar forms, for example, or are those matrices different, whether something else would work for us. Or the whole world of risk assessment could be opened up to us from a little different scope.”*

In connection with the development of communication, in 17% of the interviews’ responses, participants mentioned that repeating information improves the person’s recollection. In 12% of the interviews categorized as “Other”, the respondents could not give any development proposal. The activities and discussions of the team as well as clear guidance and resources were mentioned in 10% of the interviews.

## 4. Discussion

In this study, the OHS risk assessment skills were examined. The Delphi survey provided information gathered from OHS experts on what they considered to be the important OHS risk assessment skills of people working in different roles. The role of safety experts in research extensively covers the need for safety management and related training, but there are few actual competence criteria related to purely risk assessment [48,49,50]. The present study shows that the experts agreed that the most important issue for an employee’s OHS risk assessment skills is identifying the hazards of their own work and minimizing the associated risks. According to the literature, various methods have been developed to identify hazards, such as using visual hazard identification and transmission boards [12,19,25].

The second most important requirement for employee’s OHS risk assessment skills was being able to identify situations in which work needs to be suspended. According to the Finnish Occupational Safety and Health Act [5], an employee has the right to refrain from doing work that poses a danger to their own health or the health of others. The employee is also obliged to notify the employer that they have eliminated or rectified the defect or deficiency. Wang et al. [51] have investigated the safety risks posed by high frequency and low severity that could be avoided if corrective action is taken in a timely manner. In two of the companies included in this study, the nature of the work was such that unsafe work was prevented by redesigning the work plan.

Regarding the skills of supervisors in the OHS risk assessment, “influencing by one’s own example and attitude” was considered to be the most significant by experts. This was somewhat highlighted in the interviews when participants discussed different safety sessions and discussions and reviewed risk assessments with the employees involved. In addition, improving the understanding of risk assessment was mentioned as a development measure, which also affects the supervisor’s motivation. This is in line with the study by Huang et al. [52], which emphasized that when a supervisor leads by example, they encourage subordinates to maintain a safer workplace. It should be noticed that employee’s and supervisor’s skills include more specific factors than OHS experts. For the OHS expert, the most important risk assessment skills were related to understanding and managing the wholeness, concepts and principles of the risk assessment. As the quality of risk assessment is dependent on many steps [16], it is important that certain persons have a holistic view of the risk assessment.

In the interviews, the most important guidelines for assessing the hazards of one’s own work were considered to be daily activities, which is in line with previous studies, as safety management should be a daily activity in which supervisors and managers lead by example and encourage their subordinates to work safely [52]. However, Jeschke [53] has revealed a major conflict between managers’ daily work and safety, which they viewed as an extra task. In this study, it was found that, in the workplaces, employees are encouraged to make safety reports and do various safety rounds, sessions, or walks. In addition, incidents are regularly reviewed under the supervision of a supervisor. The last-minute hazard assessment emerged as another form of guidance in the interviews. Both the memory card method and the silent self-assessment of work hazards were used.

In this study, the interviewees mainly named written instructions, on-the-job learning and training as methods of current orientation, training, and guidance. In addition to traditional training methods, interviewees mentioned, among other things, training via MS Teams, video training and e-learning. Based on previous studies, the use of different training methods in developing skills has been highlighted [23,24]. For example, COVID-19 has caused changes in the organization in terms of teaching and accelerated the possibilities of distance learning [54]. Although it is often assumed that skills are developed in training programs, especially via textbook- and computer-based methods, previous research has shown that this is not a certainty. Training with both safe and high-risk examples has shown the highest accuracy and may lead to fewer under- or overestimations of risk in the assessment [32]. However, this study revealed an experience of not receiving any risk assessment-related orientation. In addition, companies did have written instructions and guidelines but a large proportion of respondents were uncertain about the instructions, felt the amount of instructions was low, or were unsure where to find the instructions. Furthermore, it was felt that although support is available, one must be able to take the initiative and ask for support from the right party.

Further study is needed to explore the effective intervention for training OHS risk assessment skills. In the current study, the experts thought that training and coaching are among the most feasible methods of developing risk assessment skills. Burke et al. [30] suggested enhancing learning through activity, dialogue and reflections. Ho and Dzeng [29] revealed that an e-learning method can be effective if the learning environment motivates the learner. In this study, the interviewees also raised education as the most significant way to develop OHS risk assessment skills. Suggested training methods included training given by a person outside of the organization, self-learning on the computer and mentoring. The OHS experts said that another feasible method of developing employees’ risk assessment skills is having clear guidance. Supporting this, a study by Bruhn and Frick [55] showed how a project can fail with under- or oversteering as well as with overly general or overly detailed guidance. Furthermore, in this study, the interviewees suggested improving risk assessment understanding and reviewing different perspectives.

Huang et al. [52] emphasized the importance of supervisors having a comprehensive understanding of safety rules and raised the need for communicating well with their employees. The current study made similar findings [52]; there is a need to improve communication between management and employees. Furthermore, the results of risk perceptions among employees and assessors differ, but the situation can be improved by using information strategies that simplify learning and improve risk perception [56]. Further research should be focused on how to make existing instructions and guidance better known to workers. Important questions include, why does one person easily find the right instructions and another feels that the instructions are unclear? Likewise, the employees’ experience of their own activity in obtaining support also raises questions, so how much can the support depend on the person? The focus could be on the communication and communications methods by studying various case companies.

### Limitations

The companies that participated in this study developed their own OHS management systems long before this study. Consequently, the results of the study may not be directly generalizable to companies with a lower level of safety. Furthermore, the reference framework for this study was European OSH legislation [6] and its national Finnish Occupational Health and Safety Act [5]. As this is an EU Framework Directive, it sets minimum standards risk assessment and hence, the results are generalizable to European countries at some level. As the Delphi survey and interview process are explained in detail with explanations about the demographics of the participants, similar research can be carried out by other researchers in other countries. Further studies are recommended in multiple companies under different conditions, for example micro and small companies, in order to further generalize the results.

The other limitations of this study are the response rate to the Delphi survey and the limited number of interviewees (*n* = 53) in the qualitative studies as well as the interpretation of these studies. A Delphi survey was e-mailed to the OHS specialists and managers, whose response rates varied [57,58]. By sending a reminder email each round, the number of responses increased. Small variations in the questionnaire questions or in the words used may affect the results obtained; consequently, in the second round, organizational factors were asked in addition to individual ones [47]. The results of this study were supported by the fact that the number of respondents increased in each round: 8 respondents in the first round, 12 respondents in the next round and 13 respondents in the last round. 

Some of the interviews were conducted under time pressure, and in some of the companies interviewed, participants were supervisors, managers, and occupational safety and health representatives with no employees. However, the ratio of employees’ positions was overall fairly uniform. Although the interviewees had different education levels, work experience and backgrounds, similar issues began to emerge from the responses, so it can be said that this study used enough interviews. The design of the interviews took into account the interaction between the researchers and the interviewees. The interview questions were shown as text and spoken aloud. The semi-structured interview ensured that all themes were discussed, but it also provided room for more in-depth and free discussion [59]. This was done to make the interviewees feel comfortable and ensure that the discussion stayed on topic [42]. Similarly, some pitfalls and problems were taken into account when conducting a qualitative interview [60]. Efforts were made to improve the reliability of the study in a number of ways. The interviews were conducted in collaboration with two researchers. After each interview, the notes were reviewed and a separate file was compiled from them. The material was mainly coded by the same two researchers, who discussed the meaning of citations and codes [61]. The coding was iteratively carried out, reviewing the citations of a particular code if necessary and ensuring that they corresponded to the code [62]. In addition, the theme design was done by two researchers. 

## 5. Conclusions

The results of this study are in line with those of previous studies. The most important issues related to the risk assessment skills of the employees and supervisors were unanimously determined to be identifying hazards and minimizing the risks of one’s own work for employees and the supervisors influencing of their subordinates through their own example and attitude. Understanding and managing the wholeness of safety practices and understanding and managing the meaning, concepts, and criteria of risk assessment as well as the related workplace safety legislation were the most important risk assessment skills for the OHS experts.

The interviews were used to determine the most significant ways to support risk assessment skills. Currently, learning at work and training as well as written instructions are the most often used support methods. However, there were some uncertainties about the instructions and feelings of not receiving enough orientation, guidance and support. Interviewees felt that in order to receive the support, they have to be the active party.

For the development of risk assessment skills, the survey provided expert opinions on the most feasible ways to support risk assessment skills, such as training and coaching, clear instructions, as well as suggestions from interviews on how risk assessment skills can be supported, such as via various training methods. Training methods included video teaching and training provided by a person outside the organization. In addition, the need to increase understanding of risk assessment and utilizing different perspectives were highlighted.

The results of this study can be used to find the most import development needs in order to create concrete development plans to improve risk assessment skills. Based on a Delphi survey, companies receive information on what OHS experts consider important risk assessment skills for personnel in different positions. Furthermore, this study presents how risk assessment skills are currently supported and suggests ways to further support and develop the risk assessment skills. Knowledge of the current situation can enable a company to choose the best development actions and implementations. Hence, it is recommended that companies perform a similar survey and interviews to determine their personnel’s need and receive feedback on risk assessment skills and support.

## Figures and Tables

**Table 1:** Background information of the Delphi survey (*n* = 17).

Companies and Stakeholders	Industry	Delphi Surveys Responders Per Company/Authority
Company A	Manufacturing	2
Company B	Electrical power generation transmission and distribution	1
Company C	Transportation and storage	3
Company D	Manufacturing	3
Company E	Other technical testing and analysis	2
Company F	Public authority	4
Company G	Consulting business	2

**Table 2:** Background information about the interviews (*n* = 41) and interviewees (*n* = 53).

Companies	Industry	No. of Interviews and Interviewees (*n* = 41;53)
Company A	Manufacturing	6;9
Company B	Electrical power generation, transmission and distribution	7;7
Company C	Transportation and storage	17;19
Company D	Manufacturing	9;11
Company E	Other technical testing and analysis field	2;7

## Appendix A

**Table A1:** The number and percentage of responses to the themes as well as the number and percentage of interviews in which the theme is mentioned.

	The Number of Themes		The Number of Interviews in Which the Theme Was Mentioned	
	*n*	%	*n*	%
**How is the conducting of risk assessments instructed?**				
Written instructions	34	44	23	56
Unsure of guidance	18	23	17	41
Training	17	22	14	34
Guidance provided by a particular person	7	9	7	17
Unwritten instructions	1	1	1	2
**Have you/others involved in the risk assessment received orientation about it? If so, what kind?**				
Learning at work	37	33	23	56
Training	33	30	24	59
Employee feels that there was no orientation	31	28	23	56
Other	10	9	8	20
**How are individual employees instructed to assess the risks of their own duties?**				
Everyday activities	20	51	19	46
Last-minute risk/hazard assessment	12	31	12	29
One’s own activity	3	8	3	7
Not instructed	2	5	2	5
Oral work permit	2	5	2	5
**How are risk assessment skills currently supported?**				
One’s own activity	42	49	26	63
Training and guidance	22	26	17	41
Unsupported	12	14	12	29
Guided discussions	11	13	9	22
**In what ways could the risk assessment skills be improved?**				
Training activities	42	45	26	63
Improving understanding of risk assessment	16	17	13	32
Different perspectives	13	14	10	24
Flow of information	10	11	7	17
Other	5	5	5	12
Activities of the team	5	5	4	10
Guidance and resources	4	4	4	10
